# Supplementary material for: Treponema denticola as a prognostic biomarker for periodontitis in dogs
Source: PLoS One. 2022 Jan 21;17(1):e0262859. doi: 10.1371/journal.pone.0262859 (PMC8782364; doi:10.1371/journal.pone.0262859)

**Supporting Figure S1.** Test results of qPCR sensitivity with the primer/probe set

a) For detection of *Aa*

| No. | Template DNA | Copy number | *Ct* value |
| --- | --- | --- | --- |
| 1 | STD 1 for Aa (10^-2^) | 7.14E+08 | 14.11 |
| 2 | STD 2 for Aa (10^-3^) | 7.14E+07 | 17.35 |
| 3 | STD 3 for Aa (10^-4^) | 7.14E+06 | 20.86 |
| 4 | STD 4 for Aa (10^-5^) | 7.14E+05 | 24.05 |
| 5 | STD 5 for Aa (10^-6^) | 7.14E+04 | 27.31 |
| 6 | STD 6 for Aa (10^-7^) | 7.14E+03 | 30.99 |


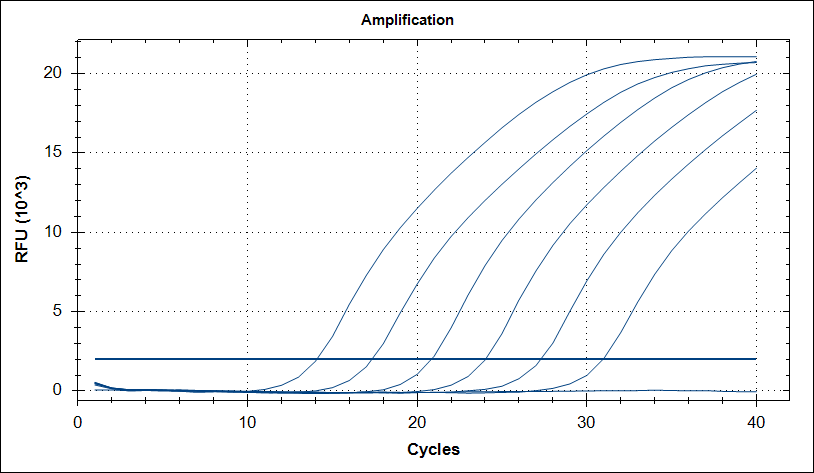


b) For detection of *Pg*

| No. | Template DNA | Copy number | *Ct* value |
| --- | --- | --- | --- |
| 1 | STD 1 for Pg (10^-2^) | 1.41E+09 | 14.39 |
| 2 | STD 2 for Pg (10^-3^) | 1.41E+08 | 17.68 |
| 3 | STD 3 for Pg (10^-4^) | 1.41E+07 | 21.01 |
| 4 | STD 4 for Pg (10^-5^) | 1.41E+06 | 24.39 |
| 5 | STD 5 for Pg (10^-6^) | 1.41E+05 | 27.73 |
| 6 | STD 6 for Pg (10^-7^) | 1.41E+04 | 31.12 |


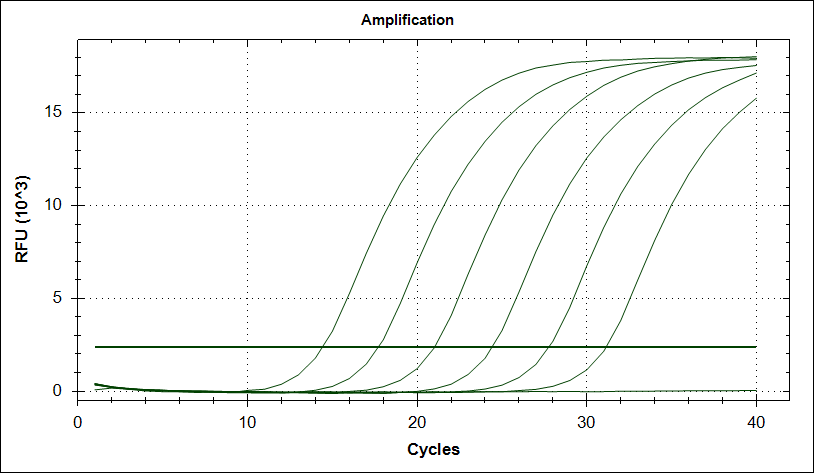


c) For detection of *Tf*

| No. | Template DNA | Copy number | *Ct* value |
| --- | --- | --- | --- |
| 1 | STD 1 for Tf (10^-2^) | 7.12E+08 | 13.80 |
| 2 | STD 2 for Tf (10^-3^) | 7.12E+07 | 17.12 |
| 3 | STD 3 for Tf (10^-4^) | 7.12E+06 | 20.43 |
| 4 | STD 4 for Tf (10^-5^) | 7.12E+05 | 23.88 |
| 5 | STD 5 for Tf (10^-6^) | 7.12E+04 | 27.28 |
| 6 | STD 6 for Tf (10^-7^) | 7.12E+03 | 30.69 |


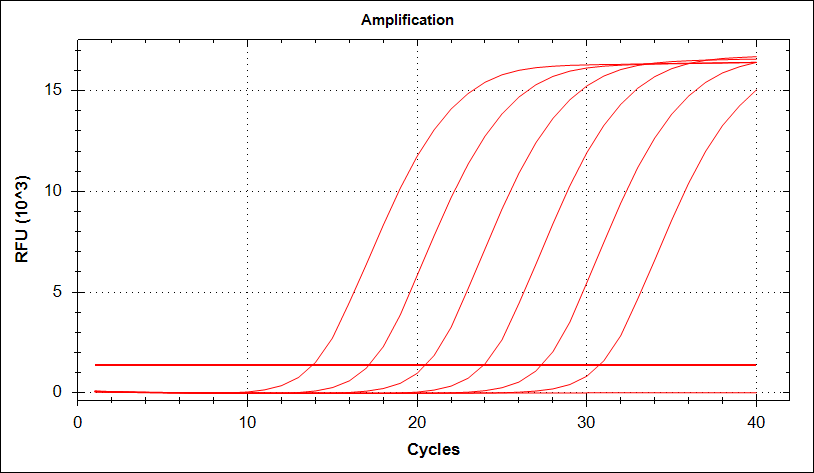


d) For detection of *Td*

| No. | Template DNA | Copy number | *Ct* value |
| --- | --- | --- | --- |
| 1 | STD 1 for Td (10^-2^) | 1.47E+09 | 14.09 |
| 2 | STD 2 for Td (10^-3^) | 1.47E+08 | 17.48 |
| 3 | STD 3 for Td (10^-4^) | 1.47E+07 | 21.28 |
| 4 | STD 4 for Td (10^-5^) | 1.47E+06 | 24.54 |
| 5 | STD 5 for Td (10^-6^) | 1.47E+05 | 28.07 |
| 6 | STD 6 for Td (10^-7^) | 1.47E+04 | 31.46 |


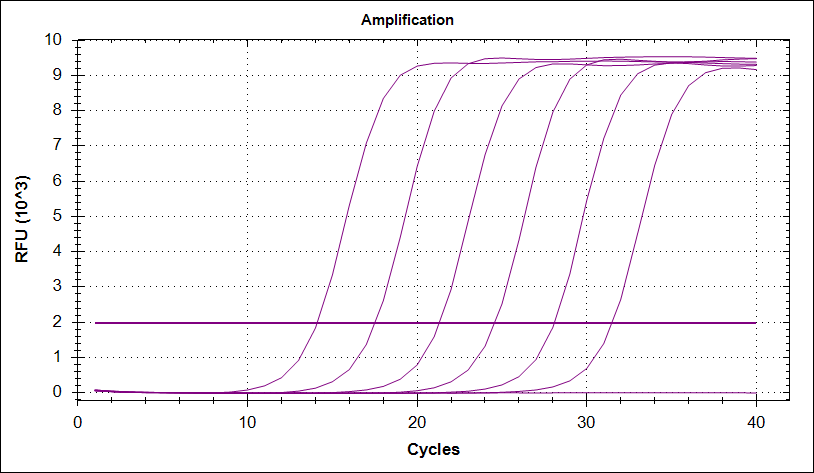


e) For detection of *Fn*

| No. | Template DNA | Copy number | *Ct* value |
| --- | --- | --- | --- |
| 1 | STD 1 for Fn (10^-2^) | 8.13E+08 | 12.51 |
| 2 | STD 2 for Fn (10^-3^) | 8.13E+07 | 15.91 |
| 3 | STD 3 for Fn (10^-4^) | 8.13E+06 | 19.35 |
| 4 | STD 4 for Fn (10^-5^) | 8.13E+05 | 22.79 |
| 5 | STD 5 for Fn (10^-6^) | 8.13E+04 | 25.95 |
| 6 | STD 6 for Fn (10^-7^) | 8.13E+03 | 29.35 |


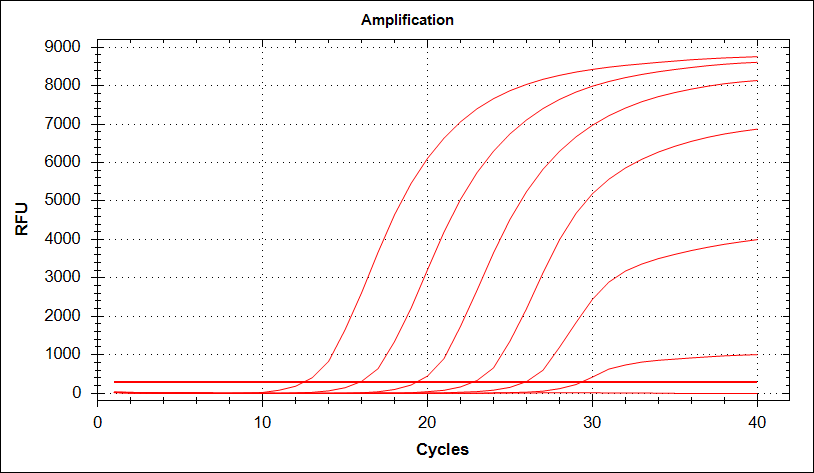


f) For detection of *Pn*

| No. | Template DNA | Copy number | *Ct* value |
| --- | --- | --- | --- |
| 1 | STD 1 for Pn (10^-2^) | 1.04E+09 | 13.63 |
| 2 | STD 2 for Pn (10^-3^) | 1.04E+08 | 16.96 |
| 3 | STD 3 for Pn (10^-4^) | 1.04E+07 | 20.30 |
| 4 | STD 4 for Pn (10^-5^) | 1.04E+06 | 23.64 |
| 5 | STD 5 for Pn (10^-6^) | 1.04E+05 | 26.93 |
| 6 | STD 6 for Pn (10^-7^) | 1.04E+04 | 29.83 |


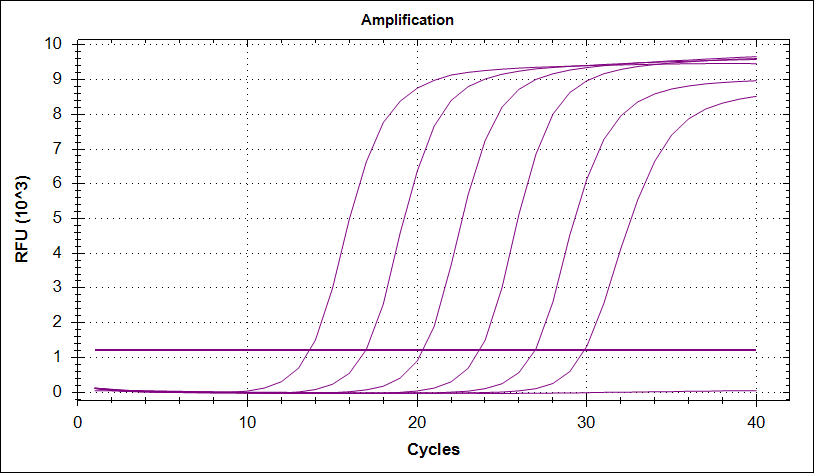


g) For detection of *Pi*

| No. | Template DNA | Copy number | *Ct* value |
| --- | --- | --- | --- |
| 1 | STD 1 for Pi (10^-2^) | 8.64E+08 | 13.29 |
| 2 | STD 2 for Pi (10^-3^) | 8.64E+07 | 16.57 |
| 3 | STD 3 for Pi (10^-4^) | 8.64E+06 | 20.09 |
| 4 | STD 4 for Pi (10^-5^) | 8.64E+05 | 23.19 |
| 5 | STD 5 for Pi (10^-6^) | 8.64E+04 | 26.81 |
| 6 | STD 6 for Pi (10^-7^) | 8.64E+03 | 29.64 |


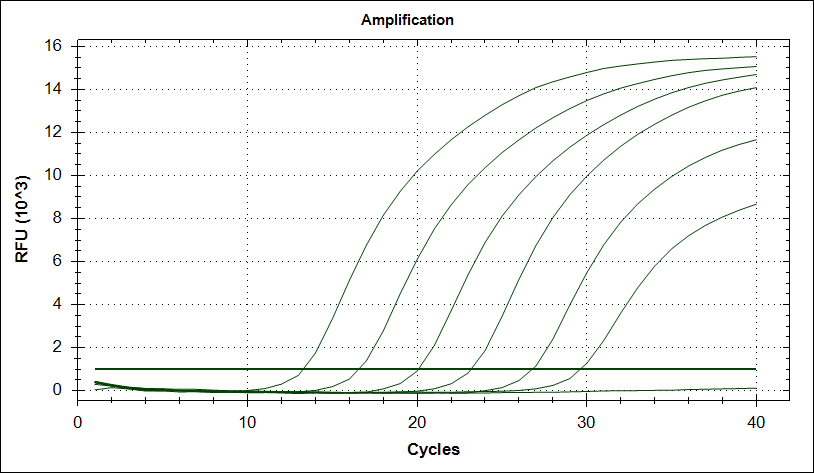


h) For detection of *Pm*

| No. | Template DNA | Copy number | *Ct* value |
| --- | --- | --- | --- |
| 1 | STD 1 for Pm (10^-2^) | 8.01E+08 | 14.09 |
| 2 | STD 2 for Pm (10^-3^) | 8.01E+07 | 17.26 |
| 3 | STD 3 for Pm (10^-4^) | 8.01E+06 | 20.68 |
| 4 | STD 4 for Pm (10^-5^) | 8.01E+05 | 23.91 |
| 5 | STD 5 for Pm (10^-6^) | 8.01E+04 | 27.30 |
| 6 | STD 6 for Pm (10^-7^) | 8.01E+03 | 30.77 |


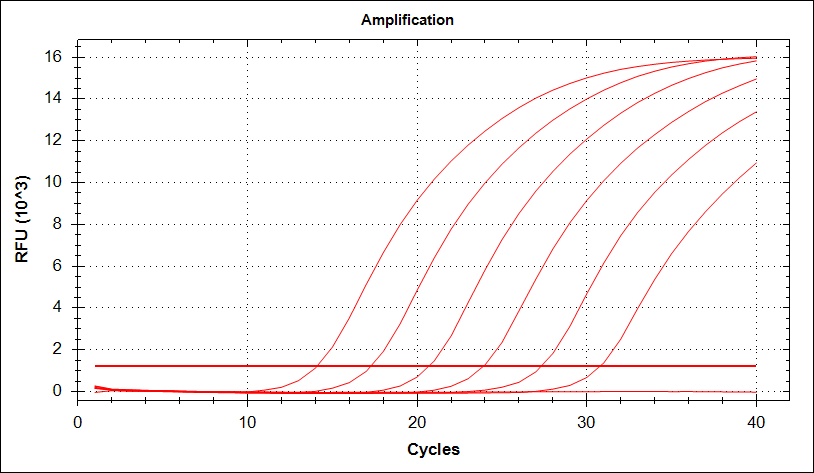


i) For detection of *En*

| No. | Template DNA | Copy number | *Ct* value |
| --- | --- | --- | --- |
| 1 | STD 1 for En (10^-2^) | 8.65E+08 | 13.71 |
| 2 | STD 2 for En (10^-3^) | 8.65E+07 | 16.76 |
| 3 | STD 3 for En (10^-4^) | 8.65E+06 | 20.14 |
| 4 | STD 4 for En (10^-5^) | 8.65E+05 | 23.50 |
| 5 | STD 5 for En (10^-6^) | 8.65E+04 | 26.75 |
| 6 | STD 6 for En (10^-7^) | 8.65E+03 | 30.19 |


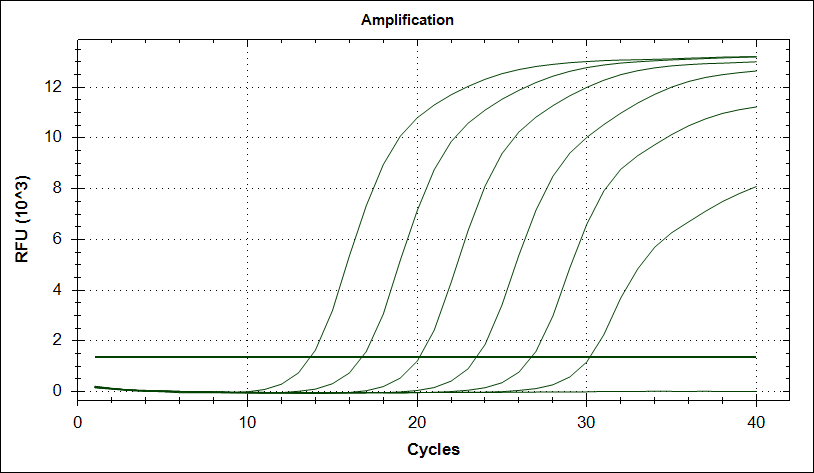


j) For detection of *Cr*

| No. | Template DNA | Copy number | *Ct* value |
| --- | --- | --- | --- |
| 1 | STD 1 for Cr (10^-2^) | 7.49E+08 | 14.13 |
| 2 | STD 2 for Cr (10^-3^) | 7.49E+07 | 17.19 |
| 3 | STD 3 for Cr (10^-4^) | 7.49E+06 | 20.45 |
| 4 | STD 4 for Cr ((10^-5^) | 7.49E+05 | 23.84 |
| 5 | STD 5 for Cr (10^-6^) | 7.49E+04 | 27.14 |
| 6 | STD 6 for Cr (10^-7^) | 7.49E+03 | 30.34 |


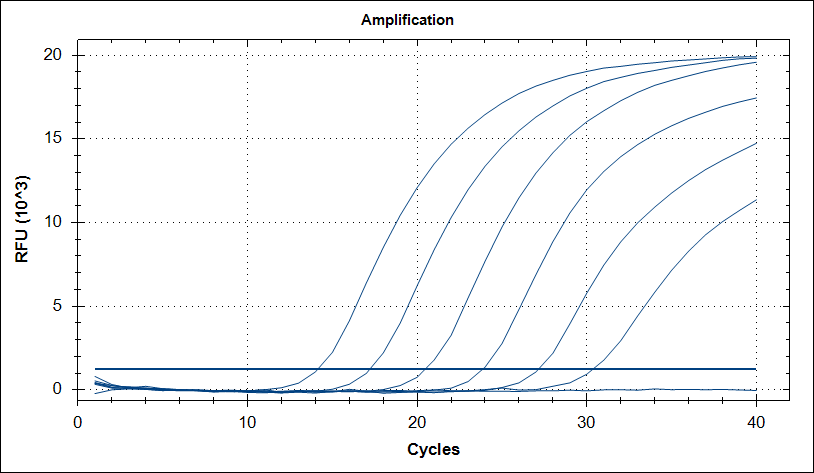


k) For detection of *Ec*

| No. | Template DNA | Copy number | *Ct* value |
| --- | --- | --- | --- |
| 1 | STD 1 for Ec (10^-2^) | 9.35E+08 | 13.76 |
| 2 | STD 2 for Ec (10^-3^) | 9.35E+07 | 16.77 |
| 3 | STD 3 for Ec (10^-4^) | 9.35E+06 | 20.22 |
| 4 | STD 4 for Ec (10^-5^) | 9.35E+05 | 23.49 |
| 5 | STD 5 for Ec (10^-6^) | 9.35E+04 | 26.73 |
| 6 | STD 6 for Ec (10^-7^) | 9.35E+03 | 30.06 |


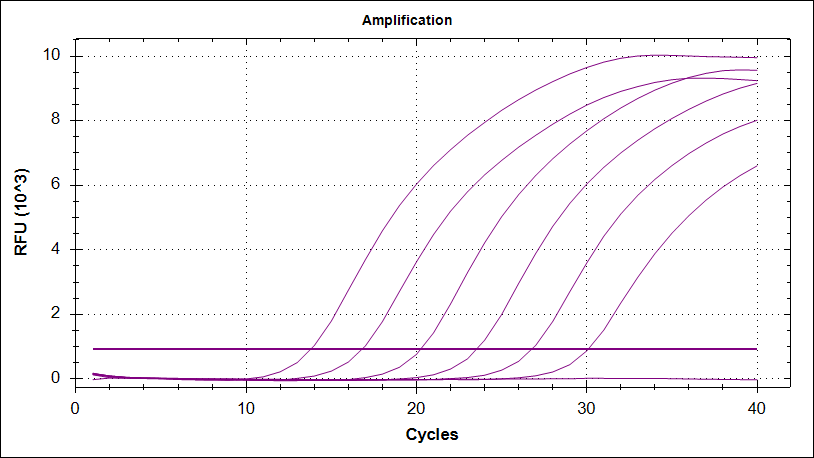

Supplement: S1 Fig — (DOCX) [file pone.0262859.s001.docx]
