# Supplementary material for: Treponema denticola as a prognostic biomarker for periodontitis in dogs
Source: PLoS One. 2022 Jan 21;17(1):e0262859. doi: 10.1371/journal.pone.0262859 (PMC8782364; doi:10.1371/journal.pone.0262859)

**Supporting Figure S2.** Standard curves generated from the *Ct* values for amplification of the target DNA of the red complex bacteria

a) *Pg* (*Porphyromonas gingivalis prtP* DNA)


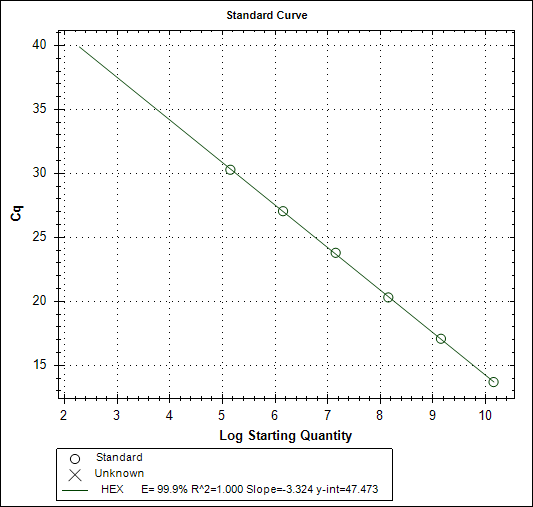


b) *Tf* (*Tannerella forsythia* karilysin DNA)


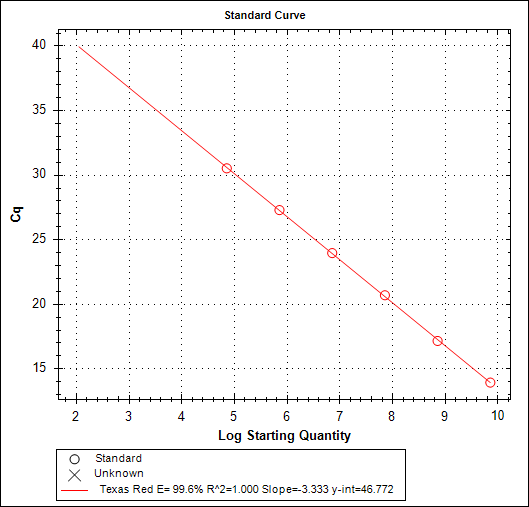


c) *Td* (*Treponema denticola opdB* DNA)


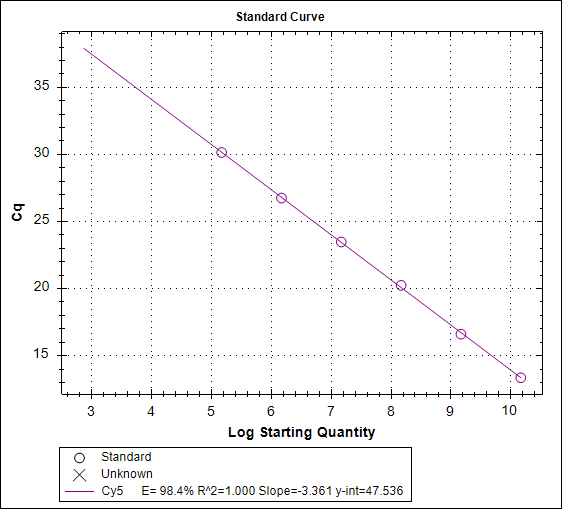

Supplement: S2 Fig — (DOCX) [file pone.0262859.s002.docx]
