# Supplementary material for: Treponema denticola as a prognostic biomarker for periodontitis in dogs
Source: PLoS One. 2022 Jan 21;17(1):e0262859. doi: 10.1371/journal.pone.0262859 (PMC8782364; doi:10.1371/journal.pone.0262859)
Supplement: S1 Table — (DOCX) [file pone.0262859.s003.docx]

Table 1. Primer and probe set used for qPCR detection of 11 bacterial species from the subgingival plaques in the teeth of dogs

| Species and  primer/probe | Primer / probe sequence (5′ to 3′) | Length  (base) | Target gene | Amplicon  size (bp) | Reference |
| --- | --- | --- | --- | --- | --- |
| *Aa*  AaLtx-F14  AaLtx-R11  AaLtx-P13 | CGGTGGAGAAGGAAATGATATTTATG  ATTGCCGTTACGCTCAAATG  FAM-CCACACTATTACGGAACATAGCGGTG-BHQ-1 | 26  20  28 | Leukotoxin (LtxA) | 139 | This  study |
| *Pg*  Pgha-F14  Pgha-R13  Pgha-P11B | GCAGGGTCAGAAAGTAACGCTC  CGATCCGTTTTACTTCACGG  HEX-CCGAGCGCAAAGAAGGCAGAA-BHQ-1 | 22  20  21 | Porphypain (*prtP*) | 80 | This  study |
| *Tf*  TfKp-F13  TfKp-R12  TfKp-P11 | CCGGCGGTTTCCTGTAGTAGA  ACTTCGTCCGTTGCAGGGTT  TEXAS RED-CTCCCTTCACCCTCTCGCCG-BHQ-2 | 21  20  20 | Karilysin protease | 68 | This  study |
| *Td*  Tdop-F13  Tdop-R13  Tdop-P01 | CATCTCTTGATGCAGCCGAAG  GTCAGGGCTTACAACATAGTCGTC  Cy5-TGGCGGAAGGAAAACAAGCC-BHQ-2 | 21  24  20 | Oligopeptidase B (*opdB*) | 98 | This  study |
| *Fn.*  FnCh-F15  FnCh-R13  FnCh-P12 | GACATCTTAGGAATGAGACAGAGATG  CAGCCATGCACCACCTGTCT  TEXAS RED-CAGTGTCCCTTCGGGGAAACCT-BHQ-2 | 26  20  22 | 16S ribosomal RNA | 73 | This  study |
| *Pn*  Pngy-F12  Pngy-R13  Pngy-P11 | GCAAGAACGTGATGACGGGA  ATTTCGCAGTCTTTGGGATCT TT  Cy5-TTGCCAGGAAAACTTGCCGA-BHQ-2 | 20  23  20 | DNA gyrase subunit B (*gyrB*) | 79 | This  study |
| *Pi*  Pipi-F12  Pipi-R13  Pi194-P13H | CCACCAACGACAACCTTCCA  TCTACTGCTTCGAGCGCAC  HEX-CAAGACAATCTCCGACGGAACGTT-BHQ-1 | 20  19  24 | Interpain A (pin0048) | 130 | This  study |
| *Pm*  PmF-30  PmR-30  Pm16S30 | AAACGACGATTAATACCACATGAGAC  ACTGCTGCCTCCCGTAGGA  TEXAS RED-TCAAAGATTTATCGGTGTAAGAAGGGCTCGC-BHQ-2 | 26  19  31 | 16S ribosomal RNA | 201 | Nonnenmacher *et al*. (2004) |
| *En*  Engl-F01  Engl-R01  Engl-P01 | ATCCACAACAAAAGCGGCCT  AGGAATGTCCGGAGCAGGAA  HEX-CAAACCAATCTGCAGCATGGG-BHQ-1 | 20  20  21 | Putative P-loop ATP-binding protein YvcJ (*glmZ*) | 157 | This  study |
| *Cr*  Crgr-F14  Crgr-R12  Crgr-P01 | GCGAAGTAGTGAGCGAAGAG  GCCTGCGCCATTTACGATA  FAM-CAAGCGTGATCATCGACAAGGATAACA-BHQ-1 | 20  19  27 | Heat shock protein (*groEL*) | 119 | This  study |
| *Ec*  EcISRF-21  EcISRR-21  EcISRP21 | AGGCGACGAAGGACGTGTAA  ATCACCGGATCAAAGCTCTATTG  Cy5-CGTGTAAGCCTGCGAAAAGCATCG-BHQ-2 | 20  23  24 | 16S-23S ribosomal RNA intergenic spacer | 69 | Price *et al*. (2007)  Modified |

***Aa****: Aggregatibacter actinomycetemcomitans,* ***Pg****: Porphyromonas gingivalis,* ***Tf****: Tannerella forsythia,* ***Td****: Treponema denticola,* ***Fn****: Fusobacterium nucleatum,* ***Pn****: Prevotella nigrescens,* ***Pi****: Prevotella intermedia*, ***Pm****: Parvimonas micra,* ***En****: Eubacterium nodatum,* ***Cr****: Campylobacter rectus,* ***Ec****: Eikenella corrodens*, **F**: Forward primer, **R**: Reverse primer, **P**: Probe.
