# Supplementary material for: Treponema denticola as a prognostic biomarker for periodontitis in dogs
Source: PLoS One. 2022 Jan 21;17(1):e0262859. doi: 10.1371/journal.pone.0262859 (PMC8782364; doi:10.1371/journal.pone.0262859)
Supplement: S2 Table — (DOCX) [file pone.0262859.s004.docx]

**Supporting Information**

**Supporting Table S1.** List of bacteria tested for the specificity of the qPCR with the primer/probe set

| No. | Bacterial species | Abbreviations | Strain |
| --- | --- | --- | --- |
| 1 | *Aggregatibacter actinomycetemcomitans* | *Aa* | ATCC 29522 |
| 2 | *Porphyromonas gingivalis* | *Pg* | ATCC 33277 |
| 3 | *Tannerella forsythia* | *Tf* | ATCC 43037 |
| 4 | *Treponema denticola* | *Td* | ATCC 35405 |
| 5 | *Fusobacterium nucleatum* | *Fn* | ATCC 25586 |
| 6 | *Prevotella nigrescens* | *Pn* | ATCC 33563 |
| 7 | *Prevotella intermedia* | *Pi* | ATCC 25611 |
| 8 | *Parvimonas micra* | *Pm* | ATCC 33270 |
| 9 | *Eubacterium nodatum* | *En* | ATCC 33099 |
| 10 | *Campylobacter rectus* | *Cr* | ATCC 33238 |
| 11 | *Eikenella corrodens* | *Ec* | ATCC 23834 |
| 12 | *Streptococcus mutans* | *Sm* | ATCC 25175 |
| 13 | *Streptococcus sobrinus* | *Ss* | ATCC 33478 |
| 14 | *Porphyromonas gulae* | *P.gulae* | ATCC 51700 |
| 15 | *Escherichia coli* | *E.coli* | DH5alpha |
