# Supplementary material for: Treponema denticola as a prognostic biomarker for periodontitis in dogs
Source: PLoS One. 2022 Jan 21;17(1):e0262859. doi: 10.1371/journal.pone.0262859 (PMC8782364; doi:10.1371/journal.pone.0262859)
Supplement: S3 Table — (DOCX) [file pone.0262859.s005.docx]

**Supporting Table S2.** Test results of qPCR specificity with the primer/probe set

| No. | Oligomer set | *Aa* | *Pg* | *Tf* | *Td* | *Fn* | *Pn* | *Pi* | *Pm* | *En* | *Cr* | *Ec* | *Sm* | *Ss* | *P. gulae* | *E. coli* | NTC |
| --- | --- | --- | --- | --- | --- | --- | --- | --- | --- | --- | --- | --- | --- | --- | --- | --- | --- |
| 1 | Primer/probe set for *Aa* | + | - | - | - | - | - | - | - | - | - | - | - | - | - | - | - |
| 2 | Primer/probe set for *Pg* | - | + | - | - | - | - | - | - | - | - | - | - | - | - | - | - |
| 3 | Primer/probe set for *Tf* | - | - | + | - | - | - | - | - | - | - | - | - | - | - | - | - |
| 4 | Primer/probe set for *Td* | - | - | - | + | - | - | - | - | - | - | - | - | - | - | - | - |
| 5 | Primer/probe set for *Fn* | - | - | - | - | + | - | - | - | - | - | - | - | - | - | - | - |
| 6 | Primer/probe set for *Pn* | - | - | - | - | - | + | - | - | - | - | - | - | - | - | - | - |
| 7 | Primer/probe set for *Pi* | - | - | - | - | - | - | + | - | - | - | - | - | - | - | - | - |
| 8 | Primer/probe set for *Pm* | - | - | - | - | - | - | - | + | - | - | - | - | - | - | - | - |
| 9 | Primer/probe set for *En* | - | - | - | - | - | - | - | - | + | - | - | - | - | - | - | - |
| 10 | Primer/probe set for *Cr* | - | - | - | - | - | - | - | - | - | + | - | - | - | - | - | - |
| 11 | Primer/probe set for *Ec* | - | - | - | - | - | - | - | - | - | - | + | - | - | - | - | - |
| 12 | Primer set for *P. gulae* | - | - | - | - | - | - | - | - | - | - | - | - | - | + | - | - |

* Genomic DNA (20 ng) was used as a template, respectively.

** NTC: Non-template control
